# Identification of L-Valine-initiated-germination-active genes in *Bacillus subtilis* using Tn-seq

**DOI:** 10.1371/journal.pone.0218220

**Published:** 2019-06-14

**Authors:** Cameron V. Sayer, Bidisha Barat, David L. Popham

**Affiliations:** Department of Biological Sciences, Virginia Tech, Blacksburg, VA, United States of America; Universite Paris-Sud, FRANCE

## Abstract

Bacterial endospores can survive harsh environmental conditions and long-term dormancy in the absence of nutrients, but can rapidly germinate under favorable conditions. In the present study, we employed transposon sequencing (Tn-seq) to identify genes with previously uncharacterized roles in spore germination. Identified genes that encoded spore inner membrane proteins were chosen for study of defined mutants, which exhibited delayed germination in several assays in response to varying germinants. Significantly slowed release of DPA indicated that mutants were affected in Stage I of germination. Several mutants exhibited phenotypic traits consistent with failure of a GerA germinant receptor-mediated response, while others appeared to have a more general loss of response to varied germinants. Use of a *gerA-lacZ* transcriptional fusion and quantitative western blotting of GerAC allowed mutants to be classified based upon normal or decreased *gerA* transcription and normal or reduced GerA accumulation. Fourteen genes were identified to have newly described roles within *Bacillus* spore germination. A more complete understanding of this process can contribute to the development of better spore decontamination procedures.

## Introduction

Bacterial endospores are capable of extended periods of dormancy while remaining resistant to a variety of chemical and physical decontamination measures [1]. Dormant spores can rapidly germinate when in a suitable environment, returning to a vegetative state [2, 3]. These factors allow endospores produced by certain species of *Bacillus* and *Clostridium* to excel as human pathogens, act as potential bioterrorism agents, and contribute to significant food contamination events [4, 5]. Preservation of dehydration of the metabolically inactive spore core is the greatest factor in spore resistance properties and maintenance of spore dormancy [1]. This dormant state is maintained by the inner spore membrane, which exists in a largely non-fluid state [6], and a thick layer of peptidoglycan termed the cortex [7]. Additionally, the accumulation of small molecule solutes within the core, such as calcium dipicolinic acid (DPA), contribute to spore dehydration and resistance properties [1].

When dormant spores sense an environment conducive to vegetative growth, they will rapidly germinate. Environmental sensing is achieved through the action of proteins expressed late in sporulation, termed germinant receptors. *Bacillus subtilis* encodes three functional Ger receptors: GerA, GerB, and GerK [8]. The GerA receptor responds to amino acids such as L-Alanine and L-Valine, while the GerB and GerK receptors work together to respond to a mixture of L-Asparagine, D-glucose, D-fructose and K^+^ ions (AGFK) [2]. The mechanism of signal transduction from Ger receptors to other spore components to initiate germination is not well understood, but a major event is the opening of a channel involving SpoVA proteins to release Ca^2+^-DPA from the spore core [9]. The GerD protein of *Bacillus* species is required for a rapid response to germinants. Recent work suggests that GerD is essential for the colocalization of Ger receptors in the spore’s inner membrane in a cluster termed the germinosome and probably plays an intermediate role in the signal transduction pathway from germinant-receptor complex to downstream germination effectors [9, 10].

Each Ger receptor is composed of three subunits: A, B, and C. The A subunits are transmembrane proteins featuring sizable domains on each side of the membrane [3, 8]. The B subunit proteins are thought to be integral membrane proteins that may be involved in germinant recognition [11]. The C subunits are lipoproteins attached to the other surface of the membrane [12]. Following triggering of the Ger receptors, water begins to partially rehydrate the spore core, and Ca^2+^-DPA is released along with other ions contained within the spore core. The spore cortex is then degraded through the action of germination-specific lytic enzymes (GSLEs) which allow the spore core to continue to expand and return to a fully hydrated state [13]. The spore will then resume metabolism and continue through outgrowth, eventually returning to a fully vegetative state.

The goals of the current study were to identify additional genes with potential roles in spore germination. Whereas previous studies have characterized genes whose loss resulted in a near complete block of germination, we sought to find genes with more subtle phenotypes that were potentially missed by previous procedures. Creation of a transposon-insertion mutant library and submission of spores produced by that library to germination conditions, in combination with Transposon Sequencing (Tn-seq)[14], facilitated identification of 61 genes that exerted significant effects on germination efficiency or rate. Among these, 14 genes had not been previously associated with spore germination and had been shown to produce proteins within the spore membrane proteome, and these were selected for further characterization. Defined gene knockout strains demonstrated reduced germination. Further studies implicated certain genes in affecting the overall GerA receptor abundance within the dormant spores.

## Materials and methods

### Strain constructions

All strains are listed in Table A in S1 File. DNA extracted from a previously constructed Tn-insertion library [15] was transformed into PS832 with selection for spectinomycin resistance (100 μg/mL). Roughly 150,000 independent transformants were pooled from plates to produce a new library.

Mutants lacking single genes were obtained from the *Bacillus* Genetic Stock Center. Each mutation was a deletion/insertion, with the gene of interest replaced by an erythromycin resistance gene flanked by two *loxP* sites [16]. The mutations were introduced into *B*. *subtilis* strain PS832 by natural transformation with selection for erythromycin (2.5 μg/ml) and lincomycin (12.5 μg/ml) (MLS) resistance.

Chromosomal DNA from *B*. *subtilis* strain DPVB724 with a *gerB* deletion and insertion of a chloramphenicol (3.0 μg/ml) resistance gene was used to transform strains to GerB^-^. This was done to reduce background detection of GerBC during Western blot quantification of GerAC.

*B*. *subtilis* strain DPVB761 with a *gerA-lacZ* fusion marked with an MLS resistance gene was obtained from the Setlow lab [17, 18]. Since all the putative Ger mutant strains had the same resistance gene marker, it was essential to delete the MLS resistance gene in order to transform the mutant strains with chromosomal DNA carrying the *gerA-lacZ* fusion. The Cre recombinase was expressed from plasmid pDR244 [16] to stimulate deletion of the resistance gene, leaving an unmarked in-frame deletion mutation. The *gerA-lacZ* fusion was then introduced by natural transformation into the 14 mutant strains carrying an unmarked deletion, with selection for MLS resistance.

Chromosomal DNA from *B*. *subtilis* strain DPVB833, in which *gerA* expression is under the control of spore-specific promoter *PsspD [19],* was introduced by natural transformation into strains with an unmarked deletions, with selection for MLS resistance. All mutations were verified by PCR and agarose gel electrophoresis.

### Spore preparation

*B*. *subtilis* spores, both single strains and the Tn-insertion strain library, were prepared in 2xSG broth [20]. Spores were harvested after 3–4 days incubation at 37°C and washed by shaking in water at 4°C and repeated centrifugation for several days. Purified spores were examined by microscopy and were judged to be >95% phase-bright spores prior to further assay.

To prepare germinated spores from the Tn-insertion library, a 10-ml suspension of dormant spores at an optical density at 600 nm (OD_600_) of 100 (~3x10^11^ spores in total) in water was heat-activated at 70°C for 30 min and cooled on ice for 10 min. The spores were then submitted to germination conditions in 50 mM NaPO_4_ buffer (pH 7.4) with 10 mM L-Valine at 37°C for 45 minutes. OD_600_ was monitored to ensure progression through germination. Germinated spores were collected by centrifugation at 12,000 *g* for 5 min at 4°C. After germination, subpopulations of dormant and partially germinated spores (𝛿≥1.25 g/ml) and fully germinated spores (𝛿~1.19 g/ml) were collected following centrifugation through a layer of 43% sodium diatrizoate.

### Sequencing of Tn insertion sites

All spore samples were decoated using urea, SDS, and DTT as described previously [21]. DNA was extracted from decoated spores using the Gram-Positive protocol from the Qiagen Blood and Tissue kit. DNA was digested with *Mme*I and quantified using Qubit (ThermoFisher). Samples were sent to the High-Throughput Sequencing and Genotyping Center at the University of Illinois for library preparation and sequencing. Adaptor ligations were performed including index bar codes as well as flow cell sequences. Following adaptor ligation, samples were PCR amplified for 18 cycles. After amplification, samples were purified and sequenced using an Illumina Hi Seq 2500, yielding reads with 5’ transposon sequence followed by a 16-bp region of genomic DNA. Sequencing read data files were uploaded an analyzed using Geneious (version 10.0) (http://www.geneious.com, [22]). Reads were filtered and trimmed leaving only genomic sequences and mapped to the JH642 *B*. *subtilis* genome. Tables were exported from Geneious listing number of reads contained within each annotated gene. Reads within each individual data set were expressed as a function of the total number of reads per million from that sample. Once normalized, both experimental data sets, dormant and germinated, were compared against one another allowing reporting of fold change between samples. DESeq2 was used to determine p-values comparing dormant and germinated samples [23]. Genes with 2-fold higher number of reads in the dormant versus the germinated sample and significant p-values (≤0.05) were selected for further study.

### Germination assays

To quantify change in OD_600_, purified spores were heat activated at 70°C for 30 minutes, quenched on ice for 5 minutes, and diluted to an OD_600_ of 0.2 in 50 mM NaPO_4_ buffer (pH 7.4) containing L-Valine (10 mM) or AGFK (10 mM L-Asparagine, 1 mM D-Glucose, 1 mM D-Fructose, 10 mM KCl). Purified spores were heat activated at 70°C for 30 minutes, quenched on ice for 5 minutes, and stimulated to germinate by dilution to an OD_600_ of 0.2 in 2xYT (Final concentrations: 8 mg/ml Yeast Extract, 12.8 mg/ml Tryptone, 4 mg/ml NaCl). Changes in OD_600_ were monitored using a Spectronic Genesys 5 spectrophotometer.

Purified spores of strains with and without overexpressed *gerA* were heat activated at 70°C for 30 minutes, quenched on ice for 5 minutes, and diluted to OD_600_ of 0.1 in 25 mM HEPES buffer (pH 7.4). Nutrient germinants were added as described above, and changes in OD_600_ were monitored using a Tecan M200 plate reader.

To quantify non-nutrient-triggered germination, purified spores at an OD_600_ of 1.0 were germinated with 1 mM dodecylamine in 20 mM KPO_4_ buffer (pH 7·4) at 37°C. At indicated times, 1 ml of germinating spore suspensions were centrifuged 15,800 x g for 2 min. The A_270_ of the supernatant was measured to quantify DPA release, which was expressed as a fraction of the total spore DPA content. Total spore DPA was determined by boiling spores for 30 min and measuring the A_270_ of the resulting supernatant.

To measure DPA release, spores from each mutant strain were heat activated at 70°C, suspended in 25 mM HEPES buffer (pH 7.4) and submitted to germination conditions with 10 mM L-Valine at 37°C. Aliquots were taken from the germinating spores at indicated times and centrifuged at 10,000 g for 45 seconds. The spore exudate was analyzed to determine the amount of DPA released [24].

To measure release of cortex fragments, mutant spores were heat activated at 70°C, suspended in 25 mM HEPES buffer (pH 7.4) and incubated with 10 mM L-Valine at 37°C. OD_600_ was recorded and aliquots were taken from the germinating spores at indicated times. Samples were centrifuged, and the spore exudate was subjected to amino acid/amino sugar analysis as previously described [25]. Peaks representing N-acetyl muramic acid (NAM) were identified based on elution times and quantified by integration of peak areas in comparison to known standards.

Dormant spores were observed using phase-contrast microscopy prior to germination, such that there were 70–100 spores per field of view and images were collected for 10 fields per sample. Spores were then heat activated at 70°C for 30 minutes, quenched on ice for 5 minutes, resuspended in 50 mM NaPO_4_ buffer (pH 7.4) and stimulated to germinate by addition of 10 mM L-Valine for 1 hour, and observed by microscopy again. The image processing toolkit Fiji was combined with segmentation machine learning algorithms [26], in the open-source software project Trainable Weka (Waikato Environment for Knowledge Analysis) Segmentation (TWS)[27]. This prototype segmentation algorithm was used to classify phase-bright and phase-dark spores. To segment the input image data, TWS transforms the segmentation problem into a pixel classification problem in which each pixel can be classified as belonging to a specific segment or class such as phase-bright or phase-dark. A set of input pixels that has been labeled is then used as the training set for a selected classifier. Once the classifier is trained, it can be used to classify either the rest of the input pixels or completely new image data. Data were analyzed for images from 3 fields per sample.

### Assay of *gerA* transcription

*B*. *subtilis* strains with a *gerA-lacZ* transcriptional fusion were grown and sporulated at 37°C in 2xSG medium. Purified spores were chemically decoated, washed, and extracted, and 𝛽-galactosidase activity was assayed using methyl-umbelliferyl-D-galactoside (MUG) as previously described [28–30]. MUG fluorescence was measured in a microplate reader (Tecan) using excitation and emission wavelengths of 365 nm and 450 nm, respectively. Standard solutions of methylumbelliferone were prepared in the same mix of buffers in order to calibrate the fluorescence readings. The average activity of PS832 (wildtype without *gerA-lacZ* fusion) samples was subtracted from the values for all samples containing the *gerA-lacZ* fusion, and all readings were normalized to decoated spore OD_600_ values.

### Western blotting

Quantitative western blots were performed on strains carrying a *gerB* deletion to avoid cross-reactivity with the GerAC antibody. Purified dormant *B*. *subtilis* spores (~100 OD_600_ units) were decoated and proteins were extracted as previously described for western blot analysis [21]. Samples were then serially diluted with 2x SDS-PAGE sample loading buffer and *gerA gerB* or *gerD* spore protein extract. TGX Stain-Free Fast Cast premixed acrylamide solution (Bio-Rad) was used, which enabled rapid fluorescent detection of tryptophan residues in proteins directly within gels and blots. The proteins were Trp-modified after separation by a trihalo compound included in the electrophoresis gel, allowing fluorescent visualization and quantitation of proteins on gels and blots immediately after the completion of electrophoresis and transfer. The total protein load and recovery for each lane was measured as the total fluorescence intensity for each lane of the blot. This was followed by probing with anti-GerAC [30] or anti-GerD [30] antibodies via chemiluminescent western blot. Band intensity was normalized to the protein present in each lane. Biorad Image Lab 6.0 was used to perform data analysis of quantitative blots. Dilutions of 1.0, 0.5, 0.25, and 0.125 concentration were blotted. Only the 1.0 and 0.5 concentrations were found to be within the linear range of detection, and these were used for quantification. Quantitative GerAC and GerD western blots were performed in triplicate.

## Results

### Identification of mutant strains with slowed or reduced germination

Seeking to identify additional genes that contribute to spore germination, Tn-seq was used to reveal genes functioning in the early stages of germination. A library of *magellan6x* transposon insertions [15] was transformed into a *B*. *subtilis* wild type strain, PS832, that is highly efficient at spore formation and germination. An estimated 150,000 independent transformants were pooled into a library from which dormant spores were produced. A sample of this spore library was collected for Tn insertion site sequencing.

Dormant spores were heat-activated and were submitted to germination-inducing conditions with 10 mM L-Valine at 37°C for 45 minutes. A 45% drop of the starting OD_600_ was observed as an indication of germination. Following germination, a density gradient was used to separate dormant (and possibly partially germinated) spores (≥1.25 g/ml) from fully germinated spores (~1.19 g/ml)[31]. This procedure was performed using two independent dormant spore preparations.

DNA was extracted from the starting spore population and the dormant and germinated spores, and the Tn insertion sites were sequenced as described [15] and mapped to the *B*. *subtilis* genome. Sequence data is available at https://www.ncbi.nlm.nih.gov/sra/PRJNA544251. The total library was found to have 5.5 x 10^4^ unique insertions spread over 3,114 genes featuring ≥10 unique insertions per gene. The number of reads within each gene were normalized as a fraction of the total reads obtained for that sample. Normalized data sets from germinated and dormant spores were then compared against one another to determine fold change. Genes with a higher proportion of reads in the dormant population indicate a possible role in germination. DESeq2 was implemented to determine p-values to further differentiate mutant abundance between sample sets [23, 32].

In total, Tn insertions in 61 genes were found to be ≥2-fold underrepresented in the germinated spores compared to those unable to complete germination (Table 1). These included all three genes of the *gerA* operon, *gerE*, coat proteins *cotH and cotE*, and genes from the *gerP* operon, all of which have known strong effects on germination. Slightly less than half of the genes identified were known previously to have either sporulation or germination defects.

**Table 1:** Genes in which Tn insertions altered germination.

Gene	p-value^a^	Fold-change Sample 1^b^	Fold-change Sample 2^b^	Reference for Ger defect
*cotH*	2.2E-35	15.0	10.9	[33]
*gerAA*	4.4E-31	150.1	43.7	[18, 34]
*gerAC*	2.0E-29	279.9	73.9	[18, 34]
*cotE*	3.8E-24	17.0	12.0	[35]
*ygaC*	2.3E-18	4.6	4.2	
*yqfT*	3.0E-16	15.4	8.8	
*ypzK*	6.3E-16	10.1	9.7	
*yqeF*	2.0E-15	3.3	5.5	
*ymzD-ymcC*^c^	5.7E-13	2.5	2.7	
*gerPF*	5.8E-13	7.4	4.1	[36]
*safA*	1.1E-11	7.3	4.1	[37]
*pcrB*	2.3E-11	3.0	2.6	
*ylbC*	3.9E-11	4.1	2.9	
*gidA*	1.3E-10	4.2	2.8	
*gerPB*	1.0E-09	5.9	3.6	[36]
*gerPC*	3.1E-09	11.7	3.9	[36]
*nocA*	4.2E-09	2.0	2.2	
*veg*	1.1E-08	4.8	3.2	[38]
*gerE*	1.8E-08	Infinite	32.0	[34]
*ytoA*	2.2E-08	5.1	4.1	
*ytpA*	1.0E-07	5.3	4.6	
*ytpB*	1.1E-07	3.9	3.5	
*rsbW*	1.2E-07	2.2	2.5	
*yfhD*	2.8E-07	4.2	4.6	
*cotZ*	7.7E-07	2.1	3.1	[39]
*yqhL*	1.1E-06	4.3	2.5	
*kinB*	1.8E-06	1.6	1.9	
*skfC*	1.9E-06	7.9	3.7	
*skfE*	3.8E-06	8.4	3.7	
*gerAB*	5.8E-06	181.7	73.6	[18, 34]
*ymaB*	6.6E-06	1.9	2.9	
*sipT*	7.5E-06	2.5	2.8	
*skfG*	9.7E-06	3.5	2.3	
*phoR*	1.0E-05	2.5	3.0	
*cotN (tasA)*	1.5E-05	2.1	2.2	[40]
*yhbJ*	2.8E-05	1.8	1.7	
*yhaM*	3.3E-05	4.6	2.5	
*ymaF*	3.7E-05	8.1	2.8	
*yabG*	1.7E-04	2.5	2.3	[41]
*spoVID*	1.8E-04	2.7	2.1	[42]
*yqhR*	3.6E-04	2.3	2.0	
*yonF*	4.7E-04	1.8	3.0	
*spoVAF*	4.8E-04	2.2	1.8	[43]
*hfq*	6.7E-04	3.2	2.5	
*yosK*	9.2E-04	4.4	6.4	
*yozE*	9.4E-04	3.0	4.8	
*yopI*	1.1E-03	2.3	2.8	
*flgN*	1.2E-03	14.2	3.6	
*gerD*	1.2E-03	2.6	1.9	[34]
*fliW*	2.0E-03	1.9	3.4	
*yfbJ*	2.0E-03	3.1	2.3	
*ytmO*	2.1E-03	4.0	3.5	
*gerPE*	2.1E-03	6.5	1.7	[36]
*phoP*	2.3E-03	3.2	3.4	
*gerPD*	4.7E-03	8.2	3.2	[36]
*tufA*	5.2E-03	5.7	3.1	
*ispA*	5.7E-03	2.9	2.8	
*yoqL*	1.7E-02	6.1	3.7	
*dnaJ*	4.7E-02	2.5	2.9	
*yaaB (remB)*	5.0E-02	7.4	2.3	
*ytxG*	2.1E-01	2.7	2.9	
*yybT (gdpP)*	2.4E-01	7.5	2.1	

^a^ p-value determined using DESeq2 [23] comparing Dormant and Germinated sample read counts.

^b^ Fold change in read counts of Dormant/Germinated samples

^c^ Intergenic region

The identified genes that were not previously implicated in spore germination were cross-referenced against proteins found in the inner spore membrane proteome [44, 45], identifying a group of 14 genes that were studied further (Table 2). The majority of these genes were largely uncharacterized and were annotated with a wide range of putative functions. Many of the genes are not known to be expressed via sporulation-specific regulatory factors but rather are regulated by vegetative cell transcriptional controls.

**Table 2:** Genes without previously known germination role identified by Tn-seq and in spore membrane proteome.

Gene	Function	Locus structure	Regulation of expression
*dnaJ*	Protein quality control	*hrcA-grpE-dnaK-dnaJ-yqeTUV*	σ^A^, HrcA [46]
*hfq*	RNA chaperone	*hfq*	Increased protein during transition to stationary phase [47]
*pcrB*	Heptaprenylglyceryl phosphate synthase	*pcrB-pcrA-ligA-yerH*	LexA regulon [48]
*phoP*	Response regulator, phosphate metabolism	*phoPR*	σ^A^, σ^B^, σ^E^, CcpA, ScoC [49, 50]
*phoR*	Sensor kinase, phosphate metabolism	*phoPR*	σ^A^, σ^B^, σ^E^, CcpA, ScoC [49, 50]
*sipT*	Signal peptidase I	*sipT*	DegU [51]
*skfE*	Export of spore killing factor (SkfA)	*skfABCEFGH*	Spo0A, AbrB, PhoP [52–54]
*ygaC*	Unknown	*ygaCD*	
*ylbC*	Unknown	*ylbBC*	σ^F^ [55]
*yqeF*	Unknown	*yqeF*	
*yqhL*	Unknown	*yqhL*	mRNA processed by RNase Y [56]
*ytpA*	Phospholipase, Bacilysocin synthesis	*ytpAB*	σ^M^ [57]
*ytxG*	General stress	*ytxGHJ*	σ^B^, σ^H^ [58]
*yybT (gdpP)*	c-di-AMP phosphodiesterase. Functions in DNA damage and acid resistance [59]	*yybS-gdpP-rplI*	σ^A^, σ^D-^induced antisense RNA [60]

### Characterization of germination of mutant strains

**Fig 1:**
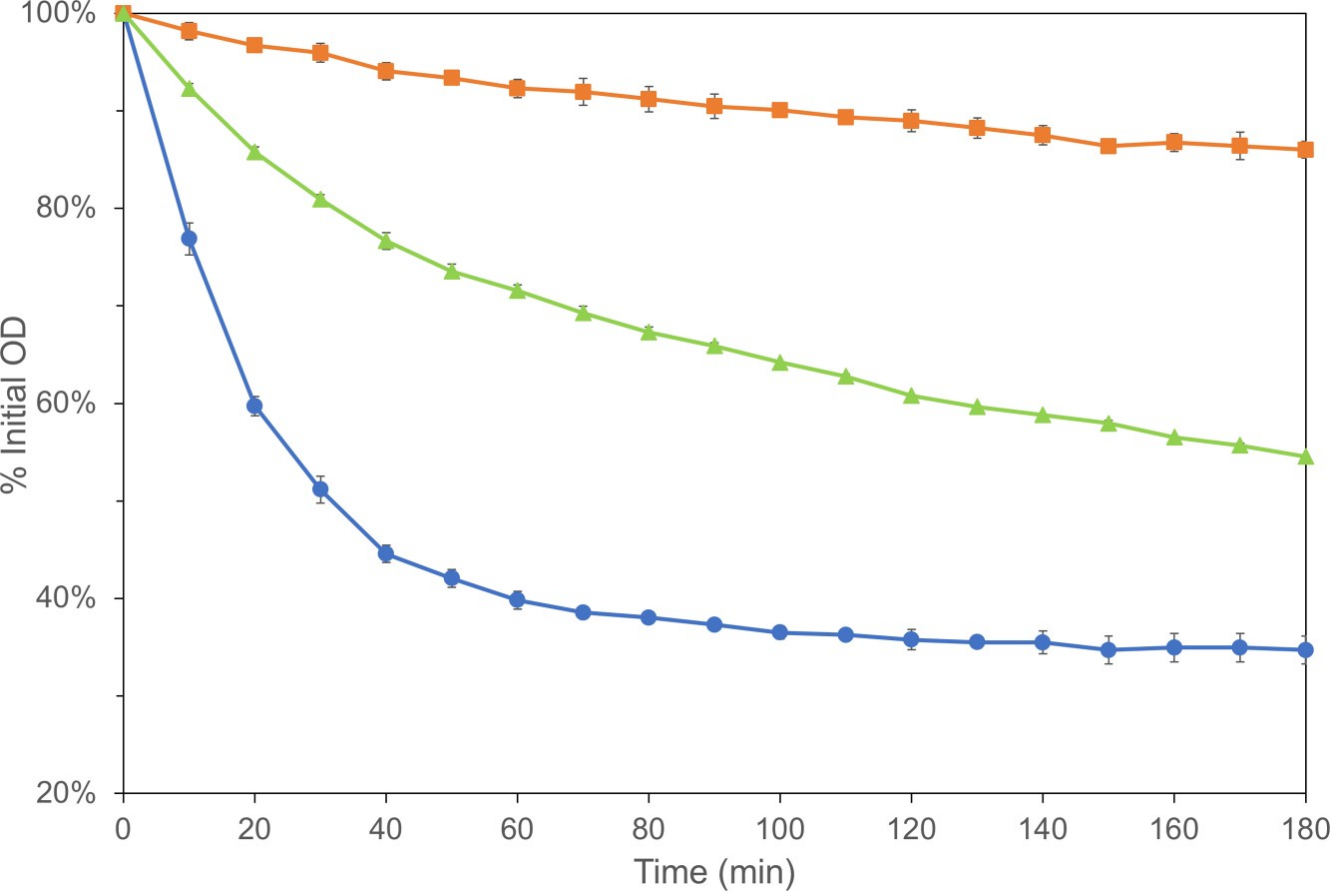
Germination rates of *B*. *subtilis* strains. Purified spores were heat activated, stimulated to germinate by addition of 10 mM L-Val, and shaken at 37°C, during which the OD_600_ was monitored. Values are averages of three assays and error bars are standard deviations. Each assay was performed on three replicate spore preparations. For the *ylbC* (■) and *phoP* (**▲**) mutants, all points after 10 min are significantly different from those of the wild type(●); P≤0.05.

Strains carrying insertion mutations [16] in each of the genes listed in Table 2 were obtained from the *Bacillus* Genetic Stock Center, and these mutations were transformed into PS832. Mutant strains were characterized with regard to growth rate and sporulation efficiency (Table 3). A number of the mutants exhibited significant growth rate defects, and the *ytxG* mutant had a severely reduced sporulation frequency. For comparison, *gerA* mutants grow and sporulate at wild type rates [34]. Purified spores were analyzed using several germination assays to verify the defects indicated by the Tn-seq data in addition to providing insight into potential function of these genes in germination. Spores were germinated with the addition of 10 mM L-Val, and OD_600_ was monitored; an example assay is shown in Fig 1 (Additional data in Figure A in S1 File). Each mutant strain exhibited a significant germination rate defect in response to L-Val in comparison to the wild type (Table 4). The most severe delays in germination rate were observed for the *ylbC*, *dnaJ*, *sipT*, and *hfq* mutants. For comparison, a *gerA* mutant exhibited <1% germination even in the presence of 200 mM L-Ala, which is more strongly stimulatory than 10 mM L-Val [61].

**Table 3:** Phenotypic properties of *B*. *subtilis* strains.

Genotype	Doubling time^a^ (min)	Sporulation efficiency^b^ (%)	DPA release^c^ (μg/ml/OD)	NAM release^c^ (nmole/ml/OD)	% phase-dark spores^d^
Wild type	20	66	5.3 ± 0.1	61.0 ± 14.2	95
*dnaJ*	31	89	1.1 ± 0.4*	25.9 ± 12.7	14
*hfq*	40	63	2.2 ± .03*	30.1 ± 6.2	48
*pcrB*	31	68	3.0 ± 0	30.3 ± 8.0	41
*phoP*	44	83	2.7 ± 0.4*	46.6 ± 5.8	63
*phoR*	35	54	3.9 ± 0.5	39.2 ± 9.5	86
*sipT*	34	54	2.3 ± 1.0*	23.5 ± 10.2*	22
*skfE*	27	59	1.8 ± 0*	40.6 ± 10.7	38
*ygaC*	21	71	3.1 ± 0.4*	36.6 ± 9.9	60
*ylbC*	29	48	1.1 ± 0.3*	14.3 ± 3.4*	19
*yqeF*	23	84	1.9 ± 0*	37.2 ± 9.4	50
*yqhL*	20	53	2.6 ± 0.4*	36.1 ± 8.1	69
*ytpA*	22	95	1.63 ± 0*	17.7 ± 2.7*	55
*ytxG*	31	18	4.8 ± 0.3	50.9 ± 4.8	90
*yybT*	21	69	4.7 ± 0.4	56.6 ± 14.1	92

^a^ Growth in 2xSG medium at 37°C

^b^ Heat-resistant count/total viable count after 24 hr incubation on 2xSG medium at 37°C.

^**c**^ Release of DPA and NAM 30 or 45 min, respectively, after exposure to 10 mM L-Val at 37°C.

* indicates a significant difference from the wild type (T-test, p<0.05). Values are indicative of averages and standard deviations of three biological replicates.

^d^ Spores pixel intensities quantified and classified as described in Materials and Methods after 60 min exposure to 10 mM L-Val at 37°C.

**Table 4:** Response of *B*. *subtilis* strains to varied germinants.

Genotype	% OD_600_ loss^a^			% DPA released by dodecylamine^b^
	L-Val (60 mins)	AGFK (60 mins)	2xYT (40 mins)	
Wild type	60 ± 1	41 ± 2	60 ± 2	75 ± 1
*dnaJ*	6 ± 0**	28 ± 10*	40 ± 3*	68 ± 4
*hfq*	26 ± 5*	30 ± 1*	52 ± 2*	75 ± 1
*pcrB*	47 ± 10*	27 ± 3*	58 ± 4	87 ± 2
*phoP*	28 ± 1*	36 ± 10	53 ± 1*	59 ± 6
*phoR*	44 ± 2*	22 ± 10*	56 ± 1	52 ± 6
*sipT*	7 ± 0**	33 ± 10	57 ± 7	69 ± 5
*skfE*	35 ± 10*	28 ± 6*	50 ± 3*	82 ± 5
*ygaC*	22 ± 3*	37 ± 10	55 ± 2	67 ± 5
*ylbC*	8 ± 1**	13 ± 2**	23 ± 8**	66 ± 3
*yqeF*	38 ± 3*	33 ± 7	58 ± 1	84 ± 1
*yqhL*	33 ± 6*	37 ± 10	54 ± 3	71 ± 2
*ytpA*	32 ± 3*	41 ± 3	47 ± 3*	73 ± 4
*ytxG*	35 ± 0*	28 ± 8	53 ± 1*	72 ± 4
*yybT*	47 ± 2*	44 ± 7	60 ± 0	76 ± 4

^a^ Values are averages and standard deviations of assays on three replicate spore preparations. OD_600_ of purified spore suspension monitored at the indicated time after addition of 10 mM L-Valine, 1X AGFK, or 2xYT while shaking at 37°C.

* indicates a significant difference (T-test, p<0.05)

** indicates a significant difference (T-test, p<0.01) from the wild type.

^b^ Values are averages and standard deviations of assays on three replicate spore preparations. DPA release by purified spore suspension monitored 100 min after addition of 1 mM dodecylamine while shaking at 37°C.

The slowed germination phenotype was further characterized by examining the individual stages of germination using assays for release of dipicolinic acid (DPA) (Stage I) and N-acetylmuramic acid (Stage II)[3]. Spores from each mutant strain were heat-activated, suspended in 25 mM HEPES buffer, and submitted to germination conditions with 10 mM L-Val at 37°C. The amount of DPA released from many of the mutant strains was vastly reduced compared to that of PS832 (Table 3). For comparison, spores of a *gerA* mutant release ≤1% of their DPA over 120 min in the presence of L-Val [62]. The only strains that were not significantly different from PS832 were the *yybT*, *ytxG*, *pcrB*, and *phoR* mutants. Mutant strains lacking *sipT*, *ylbC*, *or ytpA* demonstrated NAM release significantly less (p<0.05) than that of PS832 (Table 3, Figure B in S1 File). The rest of the mutants exhibited reduction compared to PS832 but due to high variation among replicates were not found to be significantly different (Table 3, Figure B in S1 File).

**Fig 2:**
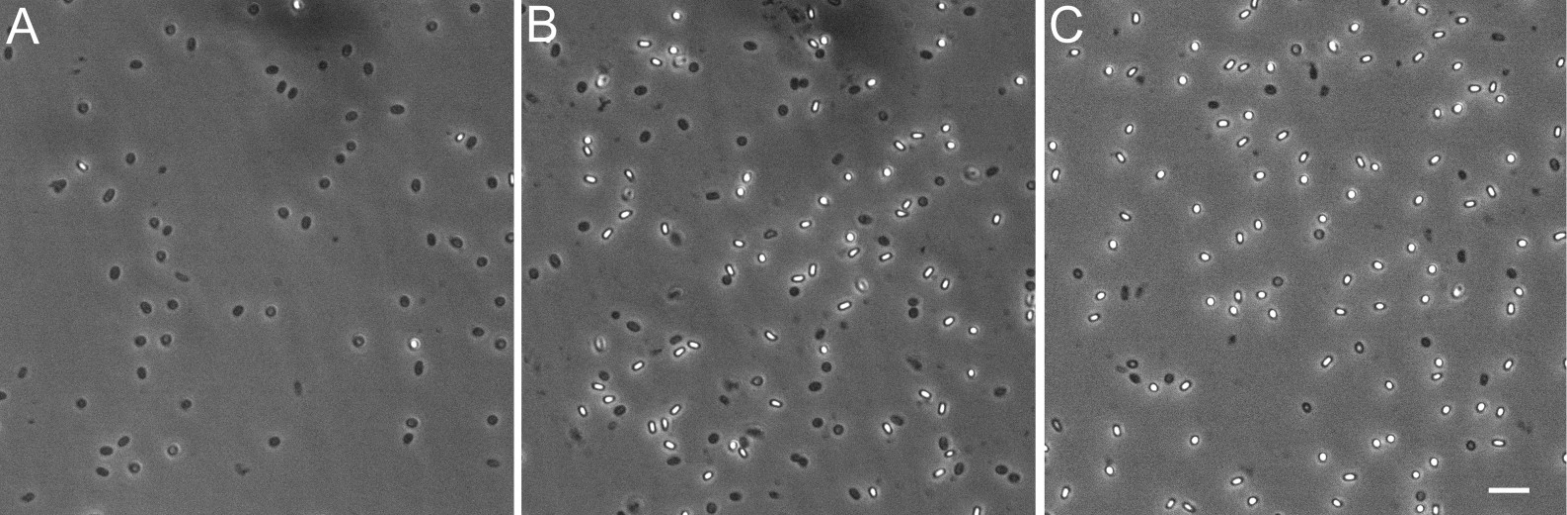
Phase-contrast microscopy of germinating *B*. *subtilis* spore populations. Purified spores of *B*. *subtilis* wild type and mutant strains were heat-activated and stimulated to germinate by addition of 10 mM L-Val followed by incubation at 37°C for 60 mins. A) PS832 B) *ytpA* mutant strain C) *ylbC* mutant strain All panels are the same magnification; the bar in panel C is 5 μm.

When mutant spore populations exhibit a decreased OD_600_ change during germination, it could result from a large percentage of the spore population germinating incompletely or from a smaller percentage of the population germinating at a normal rate with the remainder remaining fully dormant. Spores of the various strains were imaged using phase-contrast microscopy, both prior to and one-hour post-germination with 10 mM L-Val (Fig 2), and spores were classified as phase-bright or phase-dark based on pixel intensities (Figure C in S1 File). All spore samples had ≥95% phase-bright spores prior to germination. Post-germination, the wildtype spores had 95% phase-dark spores while most of the mutant strains had significantly decreased percentages of phase-dark spores (Table 3), indicating that much of the mutant strain spore populations did not initiate germination.

Additional assays were performed using the germinants AGFK and 2xYT (Table 4), which began to differentiate the mutants into distinct phenotypic groups. The first group features a reduction in germination rate to all nutrient germinants tested: L-Val, AGFK, and 2xYT, and includes strains with mutations in *skfE*, *ylbC*, *hfq* and *dnaJ*. The second phenotype includes strains with a significantly delayed L-Val germination response, via a GerA receptor, but otherwise germinate normally in response to rich medium and AGFK, via the GerB and GerK receptors. The following strains featured this phenotype: *yybT*, *ygaC*, *yqhL*, *yqeF* and *sipT*. The final group included strains with significantly slower germination rates in response to L-Val but have a delay in response to either AGFK or 2xYT, but not both. Mutants lacking *ytpA*, *phoP*, *phoR*, *pcrB* and *ytxG* feature these phenotypes. In addition, like a *gerA* mutant [63], all mutant strains were capable of normal non-nutrient, non-Ger-receptor-mediated germination, in response to dodecylamine (Table 4).

To determine if spores from mutant strains were blocked in germination or if they were simply severely delayed, spores were plated and colonies that appeared over a 48-hour period were counted. After 24 hours, all strains produced cfu/OD_600_ values similar to that of the wild type strain, and none of the strains produced a >4% increase in colonies after the first 24 hours (Table B in S1 File), indicating that the defects were a significantly slowed germination process and not death of the spores.

### Expression of the GerA receptor in mutant strains

**Fig 3:**
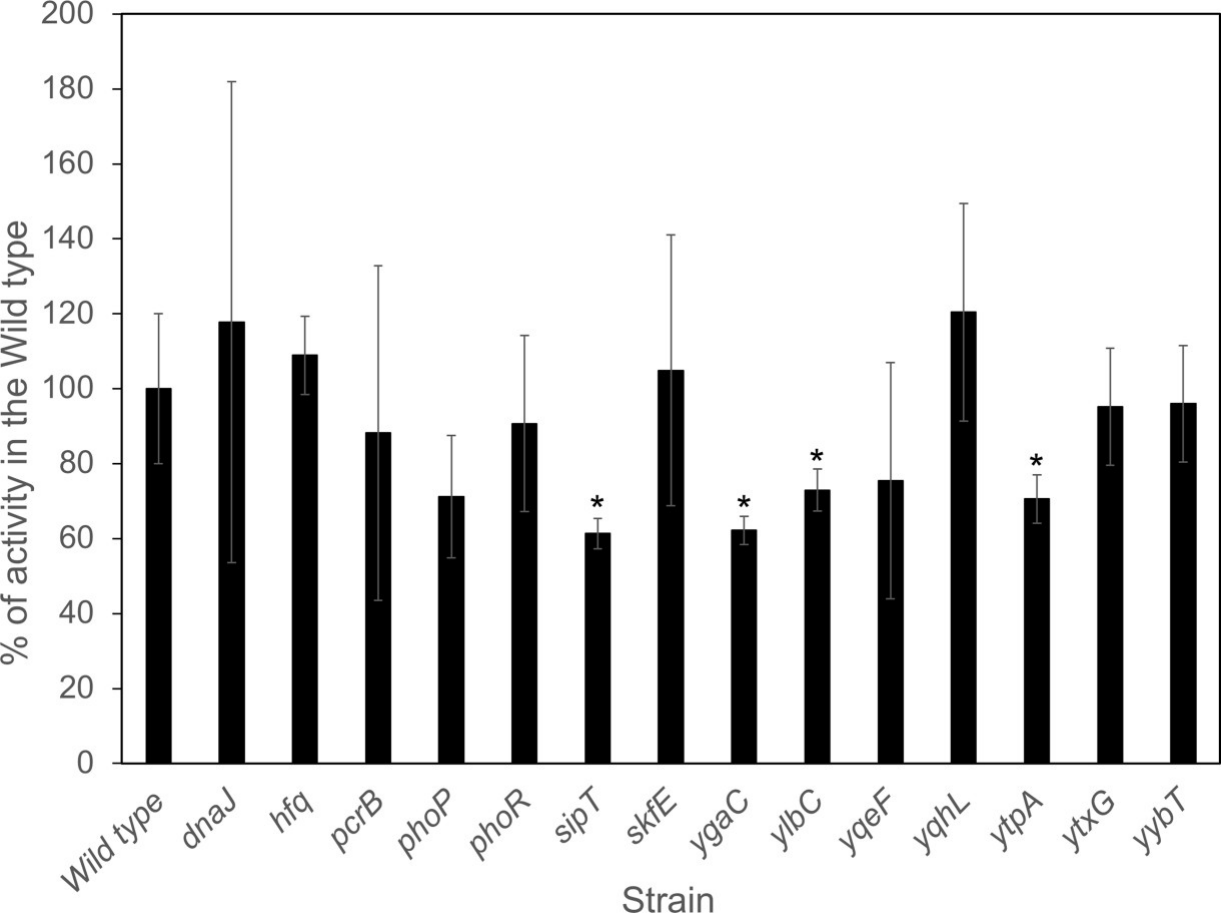
Expression of a *gerA-lacZ* transcriptional fusion. Purified spores carrying a *gerA-lacZ* transcriptional fusion were decoated and lysed, and extracts were assayed for β-galactosidase. Values are expressed as a percentage of that detected in DPVB761, the wild type strain containing the *gerA-lacZ* fusion. Values are averages of assays on three replicate spore preparations and error bars are standard deviations. * indicates a significant difference from the wild type (p ≤ 0.05).

Decreased germination in response to L-Val can result from a low abundance of the GerA receptor [29, 30]. A *gerA-lacZ* transcriptional fusion was used to determine if germination defects were correlated to reduced *gerA* transcription. Mutant strains lacking *sipT*, *ytpA*, *ylbC*, or *ygaC* showed a significant decrease in *gerA* transcription in comparison to the wildtype (Fig 3). To determine if this was a general effect on 𝜎^G^-dependent transcription, the effects of these mutation on the expression of *pbpF* and *sspB* were examined using *lacZ* fusions. The expression of these two genes was unaffected by these mutations (Figure D in S1 File).

**Fig 4:**
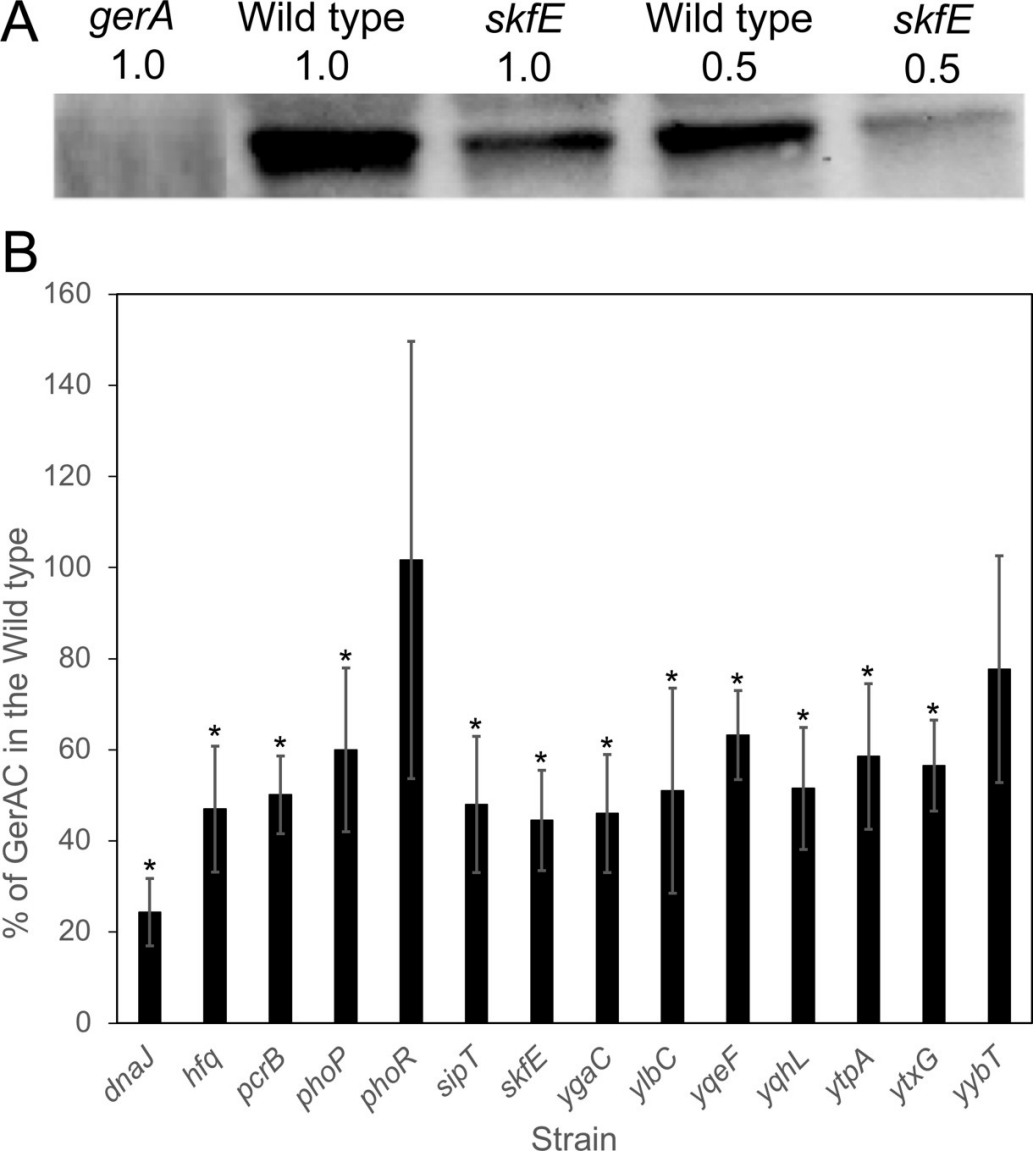
GerAC is reduced in the spores of several *B*. *subtilis* mutant strains. Equal quantities of spore suspensions were decoated and broken, and proteins were extracted, serially diluted, separated on SDS-PAGE, and transferred to PVDF membrane as described previously [21]. The membrane was probed with anti-GerAC antibodies [30] (Panel A and Figure E in S1 File). Strain genotype (All strains were also 𝛥*gerB*.) and sample dilution is indicated above each lane. Protein load and transfer to membrane in each lane was normalized as described in Materials and Methods, and the amount of GerAC detected in each strain was compared to that found in the wild type (Panel B). Error bars indicate standard deviations. * indicates a significant difference from the wild type (p ≤ 0.05).

Quantitative GerAC western blots were performed to determine the amount of GerA receptor in spores of all strains. An example Western blot is in Fig 4A, and additional blots are in Figure E in S1 File. Many of the mutant strains exhibited significant decreases in GerAC abundance; the most significant being a 75% decrease in a *dnaJ* mutant (Fig 4B). The abundance of GerD was also determined using quantitative Western blots for spores of all mutant strains, as a GerD deficiency could result in reduced germination efficiency. All the strains contained amounts of GerD similar to that of the wild type, suggesting that GerD remains unaffected in these mutant strains (Figure F in S1 File).

Overexpression of the GerA receptor in spores has previously been shown to increase the response to GerA-specific germinants [19]. A fusion of the *gerA* operon to the forespore-specific *sspD* promoter [19] was introduced into strains in order to determine if GerA overexpression could reverse the germination defects associated with the mutations under study. In almost all cases, GerA overexpression reversed the germination deficiency with L-Val (Table 5), suggesting that decreased GerA abundance made a significant contribution to the reduced germination efficiency in these mutant strains. Strikingly, in the *ytxG* mutant, overexpression of GerA increased the germination deficiency. As previously observed [19], overexpression of GerA resulted in significant decreases in the germination response to AGFK in all strains (Table C in S1 File). In mutant strains that exhibited reduced germination in response to 2xYT, GerA overexpression reversed this deficiency (Table C in S1 File).

**Table 5:** Overexpression of *gerA* suppresses germination defect of multiple mutants.

Genotype	% OD Loss^a^	
	without *sspDp-gerA*	with *sspDp-gerA*
Wild type	35 ± 4	38 ± 2
𝛥*skfE*	23 ± 5*	38 ± 3
𝛥*pcrB*	34 ± 7	37 ± 1
𝛥*ygaC*	26 ± 1*	36 ± 1
𝛥*sipT*	9 ± 5*	41 **±** 1
𝛥*ylbC*	7 ± 0*	37 ± 0
𝛥*hfq*	27 ± 3*	38 ± 3
𝛥*yqhL*	29 ± 3*	37 ± 0
𝛥*dnaJ*	12 ± 2*	36 ± 2
𝛥*yqeF*	28 ± 2*	31 ± 2
𝛥*phoR*	37 ± 1	42 ± 9
𝛥*phoP*	32 ± 1	36 ± 0
𝛥*ytxG*	25 ± 6*	15 ± 4*
𝛥*ytpA*	25 ± 0*	38 ± 2
𝛥*yybT*	37 ± 2	37 ± 2

^a^ Values are averages and standard deviations of assays on three replicate spore preparations. OD_600_ of purified spore suspension monitored 45 min after addition of 10 mM L-Valine while shaking at 37°C.

* indicates a significant difference from the wild type (T-test, p<0.05)

## Discussion

The germination and return to growth of bacterial spores is an essential step in the initiation of several diseases and of some causes of food spoilage. This Tn-seq analysis identified 42 *B*. *subtilis* genes that had not previously been associated with germination but are required for a highly efficient germination response to L-Val. As the majority of proteins previously found to play major roles in germination are membrane-associated, fourteen of these genes, whose products had also been identified in studies of the spore membrane proteome [44, 45], were further characterized. Well-defined mutations in these genes caused significantly reduced responses to L-Val, and in some cases a decreased response to other nutrient germinants. Several of these strains also exhibited slowed vegetative growth; such a growth defect could certainly alter gene expression and progression through the sporulation process, potentially affecting the germination apparatus. Future work on the specific mechanism by which these mutations alter germination may reveal such effects. For all these mutants, the germination defect appears to largely be a slow initiation of germination rather than a specific slowing of a subsequent step in the germination process. The reduced percentage of spores within the population that do initiate germination appear to progress through Stages I and II of germination at a near normal pace; rates of OD loss and phase darkening are largely mirrored by rates of DPA and NAM release. This suggests that the genes under study play a role in the earliest steps of germination initiation.

Consistent with this idea, many of the mutant strains had reduced abundance of the GerA receptor, indicating effects on receptor expression and stability or membrane incorporation. A GerA deficiency is not surprising, given that the primary screen for identification of these genes was for a reduced response to L-Val, which is recognized via GerA [2]. Based on responses to additional germinant classes, the mutant strains could be separated into distinct phenotypic groups. The first group features a reduction in germination rate with all nutrient germinants tested (L-Val, AGFK, and 2xYT), demonstrating reduced germination efficiency mediated through all receptors: GerA, GerB, and GerK. Mutant strains lacking *skfE*, *ylbC*, *hfq*, or *dnaJ* fall in this group, which also includes the strains with the greatest decreases in GerA abundance. These genes may play roles in expression or assembly of all Ger receptors or in facilitating signal transduction from germinant receptors to other parts of the germination apparatus. The well-studied function of DnaJ as a protein chaperone [64] might explain its effect on GerAC abundance in spores, and this effect suggests that DnaJ is active in the forespore late into sporulation. While the role of Hfq in Gram-positive species is not as well studied as in some Gram-negative species, its known role as an RNA chaperone [65] might exert post-transcriptional effects on the production of proteins important in the germination process. Interestingly, several other genes found in this study to affect germination (*dnaJ*, *ylbBC*, *yqeF*, *yqhL*, *yybT*) have sizable 5’ untranslated regions in their mRNAs, which might be sites for post-transcriptional regulation, or for which antisense RNAs have been identified (See http://subtiwiki.uni-goettingen.de/ for genes *yqeF*, *yqhL*, and *yybT*). A mechanism by which SkfE, which is involved in export of a sibling-killing antimicrobial [66], might affect germination is harder to imagine, but the fact that Tn insertions in two other genes in the *skf* operon also reduced germination supports the importance of this effect. Interestingly, expression of the *skf* operon is regulated by PhoPR [52], genes also implicated by this study in altering germination response.

One of the more interesting genes identified for future study may be *ylbC*, which is likely expressed as the downstream gene in the σ^F^-regulated *ylbB-ylbC* operon [55]. YlbC contains two conserved domains: an N-terminal cysteine rich secretory “CAP” domain, and a YkwD domain of unknown function that is found only in proteins of spore-forming bacteria [67]. YlbB contains two conserved CBS domains [67], which in at least one case has a role in ATP-binding [68]. Tn insertions in *ylbB* were significantly underrepresented in the germination screen (p = 0.014) but did not achieve the cutoff of a 2-fold change. Thus, *ylbB* may function with *ylbC*, but might be partially redundant with the paralogous *yhcV*, which is 𝜎^G^-dependent [55, 69] and encodes one of the most abundant transcripts in the dormant spore [70]. The mechanism by which YlbC affects *gerA* transcription and GerA abundance is a topic for ongoing study.

A second phenotypic group is composed of mutant strains with significantly delayed germination via a GerA-mediated response but germinate normally through GerB and GerK sensing. Strains lacking *yybT*, *ygaC*, *yqhL*, *yqeF*, or *sipT* exhibit this phenotype, and all except the *yybT* mutant have significantly reduced spore GerA content. The roles of these genes in germination are unclear, as most are relatively uncharacterized. YybT (GdpP) acts as a c-di-AMP phosphodiesterase and exerts pleotropic effects on physiology and gene expression [71–74]. SipT, acting as a signal peptidase [75], could certainly exert effects on assembly of membrane proteins important for germination, including GerA.

The third phenotypic group includes strains with significantly slower germination rates in response to L-Valine but either have a decreased response to AGFK or 2xYT but not both. Mutants lacking *ytpA*, *phoP*, *phoR*, *pcrB* or *ytxG* feature these phenotypes. It is not clear how or why a mutant would be deficient in GerA mediated response, have a normal GerB and GerK response, but still be deficient for germination in rich media. The PhoPR mutants have poor vegetative growth and pleotropic effects on gene expression [76, 77], which might exert quite variable effects into the sporulation process. These mutants seemed to exhibit significant variability between multiple spore preparations. Three mutants in this group may exert effects on membrane structure. YtpA is a phospholipase [57, 78], PcrB is a heptaprenylglyceryl phosphate synthase [79], and a *ytxG* mutant exhibits defects in membrane morphology [80]. Alterations in the spore inner membrane might affect assembly or function of the germination initiation apparatus. None of these three genes are specifically expressed in sporulating cells, and thus their activity levels and effects on germination might be more varied among spore preparations and possibly with regard to different germinants. Interestingly, the *ytxG* mutant was the only strain in which overexpression of *gerA* did not correct the L-Val germination defect. This overexpression in the *ytxG* mutant did decrease AGFK germination, as in other strains, suggesting that the *gerA* overexpression was successful. Perhaps a membrane defect in this mutant renders Ger protein complexes nonfunctional regardless of their expression level.

Four of the mutants identified here exhibit decreased *gerA* transcription. The predicted functions of these gene’s products provide no simple explanation for how such an effect on transcription could come about, and the mechanisms may therefore be indirect. The expression of two other 𝜎^G^-dependent genes, *pbpF* and *sspB*, was not decreased in the mutant strains, indicating that this was not an effect on the entire regulon. Altered activity of a transcription factor involved in *gerA* transcription, SpoVT or YlyA [17, 55, 69, 81, 82], could be an expected pathway for such an effect. Future work should examine the effects of these mutations on other genes within forespore-specific regulons to resolve this.

Among the germination mutants identified in our Tn-seq screen, strains that could complete Stage I of germination but were blocked in Stage II were not present. This may be due to the mutant screening process utilized. Mutants with Tn insertions in *cwlD*, which should exhibit this phenotype [83, 84], were slightly enriched in our non-germinating spore population, but not above the significance cutoff value used. Spores blocked at stage II were expected to pellet with dormant spores in the density gradient utilized [31]. One possibility is that spores blocked at Stage II were unstable through the time of incubation with germinant, density gradient separation, and subsequent washing, and thus were not efficiently recovered. Utilization of an alternative isolation method might allow identification of mutants with this phenotype.

## Supporting information

S1 File Contains Table A. *B*. *subtilis* strains used in this study; Table B. Long-term germination efficiency of *B*. *subtilis* mutant strains; Table C. Spore germination in response to diverse germinants following overexpression of *gerA*; Figure A. Germination rates of *B*. *subtilis* strains; Figure B. Release of DPA and NAM by *B*. *subtilis* strains; Figure C. Phase contrast microscopy image pixel intensities during spore germination; Figure D. Expression of 𝛔^G^-dependent genes in *B*. *subtilis* mutant strains; Figure E. GerAC is reduced in the spores of several *B*. *subtilis* mutant strains; Figure F. GerD is not reduced in the spores of *B*. *subtilis* germination mutants.
(PDF)
