# Supplementary material for: Identification of L-Valine-initiated-germination-active genes in Bacillus subtilis using Tn-seq
Source: PLoS One. 2019 Jun 14;14(6):e0218220. doi: 10.1371/journal.pone.0218220 (PMC6568419; doi:10.1371/journal.pone.0218220)
Supplement: S1 File — Contains Table A. B. subtilis strains used in this study; Table B. Long-term germination efficiency of B. subtilis mutant strains; Table C. Spore germination in response to diverse germinants following overexpression of gerA; Figure A. Germination rates of B. subtilis strains; Figure B. Release of DPA and NAM by B. subtilis strains; Figure C. Phase contrast microscopy image pixel intensities during spore germination; Figure D. Expression of 𝛔G-dependent genes in B. subtilis mutant strains; Figure E. GerAC is reduced in the spores of several B. subtilis mutant strains; Figure F. GerD is not reduced in the spores of B. subtilis germination mutants. (PDF) [file pone.0218220.s001.pdf]

S1 File

Supplemental Materials for:

Identification of Germination-Active Genes in *Bacillus subtilis* using TnSeq

Cameron Sayer, Bidisha Barat, and David L. Popham

Table A. *B. subtilis* strains used in this study.

Strain	Genotype	Source/Construction
DPVB724	Δ*gerB* Cm^R	FB72 [1]→PS832
DPVB726	Δ*gerA* Sp^R Δ*gerB* Cm^R	FB72 [1]→PS832
DPVB747	Δ*skfE* MLS^R	BKE01950[a]→PS832
DPVB748	Δ*pcrB* MLS^R	BKE06600[a] →PS832
DPVB749	Δ*ygaC* MLS^R	BKE08680[a] →PS832
DPVB750	Δ*sipT* MLS^R	BKE14410[a] →PS832
DPVB751	Δ*ylbC* MLS^R	BKE14960[a] →PS832
DPVB752	Δ*hfq* MLS^R	BKE17340[a] →PS832
DPVB753	Δ*yqhL* MLS^R	BKE24540[a] →PS832
DPVB754	Δ*dnaJ* MLS^R	BKE25460[a] →PS832
DPVB755	Δ*yqeF* MLS^R	BKE25700[a] →PS832
DPVB756	Δ*phoR* MLS^R	BKE29100[a] →PS832
DPVB757	Δ*phoP* MLS^R	BKE29110[a] →PS832
DPVB758	Δ*ytxG* MLS^R	BKE29780[a] →PS832
DPVB759	Δ*ytpA* MLS^R	BKE30510[a] →PS832
DPVB760	Δ*yybT* MLS^R	BKE40510[a] →PS832
DPVB761	*gerA-lacZ* MLS^R	PS767 [2, 3]→PS832
DPVB763	Δ*skfE* MLS^R Δ*gerB* Cm^R	DPVB724→DPVB747
DPVB764	Δ*pcrB* MLS^R Δ*gerB* Cm^R	DPVB724→DPVB748
DPVB765	Δ*ygaC* MLS^R Δ*gerB* Cm^R	DPVB724→DPVB749
DPVB766	Δ*sipT* MLS^R Δ*gerB* Cm^R	DPVB724→DPVB750
DPVB767	Δ*ylbC* MLS^R Δ*gerB* Cm^R	DPVB724→DPVB751
DPVB768	Δ*hfq* MLS^R Δ*gerB* Cm^R	DPVB724→DPVB752
DPVB769	Δ*yqhL* MLS^R Δ*gerB* Cm^R	DPVB724→DPVB753
DPVB770	Δ*dnaJ* MLS^R Δ*gerB* Cm^R	DPVB724→DPVB754
DPVB771	Δ*yqeF* MLS^R Δ*gerB* Cm^R	DPVB724→DPVB755
DPVB772	Δ*phoR* MLS^R Δ*gerB* Cm^R	DPVB724→DPVB756
DPVB773	Δ*phoP* MLS^R Δ*gerB* Cm^R	DPVB724→DPVB757
DPVB774	Δ*ytxG* MLS^R Δ*gerB* Cm^R	DPVB724→DPVB758
DPVB775	Δ*ytpA* MLS^R Δ*gerB* Cm^R	DPVB724→DPVB759
DPVB776	Δ*yybT* MLS^R Δ*gerB* Cm^R	DPVB724→DPVB760
DPVB805	Δ*skfE*	Cre expression for deletion of MLSR
DPVB806	Δ*pcrB*	Cre expression for deletion of MLSR
DPVB807	Δ*ygaC*	Cre expression for deletion of MLSR
DPVB808	Δ*sipT*	Cre expression for deletion of MLSR
DPVB809	Δ*ylbC*	Cre expression for deletion of MLSR
DPVB810	Δ*hfq*	Cre expression for deletion of MLSR
DPVB811	Δ*yqhL*	Cre expression for deletion of MLSR
DPVB812	Δ*dnaJ*	Cre expression for deletion of MLSR
DPVB813	Δ*yqeF*	Cre expression for deletion of MLSR
DPVB814	Δ*phoR*	Cre expression for deletion of MLSR
DPVB815	Δ*phoP*	Cre expression for deletion of MLSR
DPVB816	Δ*ytxG*	Cre expression for deletion of MLSR
DPVB817	Δ*ytpA*	Cre expression for deletion of MLSR
DPVB818	Δ*yybT*	Cre expression for deletion of MLSR
DPVB819	Δ*skfE gerA-lacZ* MLS^R	DPVB761→DPVB805
DPVB820	Δ*pcrB gerA-lacZ* MLS^R	DPVB761→DPVB806
DPVB821	Δ*ygaC gerA-lacZ* MLS^R	DPVB761→DPVB807
DPVB822	Δ*sipT gerA-lacZ* MLS^R	DPVB761→DPVB808
DPVB823	Δ*ylbC gerA-lacZ* MLS^R	DPVB761→DPVB809

DPVB824	Δ*hfq gerA-lacZ* MLS[R]	DPVB761→DPVB810
DPVB825	Δ*yqhL gerA-lacZ* MLS[R]	DPVB761→DPVB811
DPVB826	Δ*dnaJ gerA-lacZ* MLS[R]	DPVB761→DPVB812
DPVB827	Δ*yqeF gerA-lacZ* MLS[R]	DPVB761→DPVB813
DPVB828	Δ*phoR gerA-lacZ* MLS[R]	DPVB761→DPVB814
DPVB829	Δ*phoP gerA-lacZ* MLS[R]	DPVB761→DPVB815
DPVB830	Δ*ytxG gerA-lacZ* MLS[R]	DPVB761→DPVB816
DPVB831	Δ*ytpA gerA-lacZ* MLS[R]	DPVB761→DPVB817
DPVB832	Δ*yybT gerA-lacZ* MLS[R]	DPVB761→DPVB818
DPVB833	*PsspD::gerA* MLSR	PS3476 [4]
DPVB834	Δ*skfE PsspD::gerA* MLS[R]	DPVB833→DPVB805
DPVB835	Δ*pcrB PsspD::gerA* MLS[R]	DPVB833→DPVB806
DPVB836	Δ*ygaC PsspD::gerA* MLS[R]	DPVB833→DPVB807
DPVB837	Δ*sipT PsspD::gerA* MLS[R]	DPVB833→DPVB808
DPVB838	Δ*ylbC PsspD::gerA* MLS[R]	DPVB833→DPVB809
DPVB839	Δ*hfq PsspD::gerA* MLS[R]	DPVB833→DPVB810
DPVB840	Δ*yqhL PsspD::gerA* MLS[R]	DPVB833→DPVB811
DPVB841	Δ*dnaJ PsspD::gerA* MLS[R]	DPVB833→DPVB812
DPVB842	Δ*yqeF PsspD::gerA* MLS[R]	DPVB833→DPVB813
DPVB843	Δ*phoR PsspD::gerA* MLS[R]	DPVB833→DPVB814
DPVB844	Δ*phoP PsspD::gerA* MLS[R]	DPVB833→DPVB815
DPVB845	Δ*ytxG PsspD::gerA* MLS[R]	DPVB833→DPVB816
DPVB846	Δ*ytpA PsspD::gerA* MLS[R]	DPVB833→DPVB817
DPVB847	Δ*yybT PsspD::gerA* MLS[R]	DPVB833→DPVB818

[a] Strain obtained from the Bacillus Genetic Stock Center

Table B. Long-term germination efficiency of *B. subtilis* mutant strains[a]

Strain	Colonies after 24 hr (cfu/mL/OD)	New colonies after 48 hr (cfu/mL/OD)
Wild type	$3.5x10^8$	0
dnaJ	$9.6x10^8$	0
hfq	$3.2x10^8$	0
pcrB	$1.7x10^8$	$6.0x10^6$
phoP	$3.7x10^8$	0
phoR	$3.5x10^8$	0
sipT	$1.4x10^8$	$4.0x10^6$
skfE	$9.2x10^7$	$1.0x10^6$
ygaC	$4.0x10^8$	$1.0x10^7$
ylbC	$1.4x10^8$	$7.0x10^6$
yqeF	$1.7x10^8$	0
yqhL	$2.5x10^8$	0
ytpA	$1.8x10^8$	0
ytxG	$1.2x10^8$	0
yybT	$2.8x10^8$	$1.0x10^7$

[a] Values are from a single determination for each strain. Purified spores were serially diluted, plated on 2xSG medium, and incubated at 37°C.

Table C. Spore germination in response to diverse germinants following overexpression of *gerA*.

Genotype	% OD Loss at 60 mins with 1X AGFK		% OD Loss at 40 mins with 2xYT	
	without *sspDp-gerA*	with *sspDp-gerA*	without *sspDp-gerA*	with *sspDp-gerA*
Wild type	13 ± 4	4 ± 1.3*	34 ± 2	34 ± 1
dnaJ	6 ± 3*	1 ± 2*	23 ± 0*	29 ± 2
hfq	9 ± 5	5 ± 1*	33 ± 1	33 ± 2
pcrB	5 ± 1*	0 ± 1*	31 ± 0	36 ± 3
phoP	8 ± 3	1 ± 1*	33 ± 0	30 ± 1
phoR	5 ± 4*	1 ± 1*	32 ± 3	36 ± 4
sipT	6 ± 6*	0 ± 1*	30 ± 6	33 ± 1
skfE	4 ± 5*	3 ± 1*	32 ± 4	35 ± 0
ygaC	10 ± 1*	1 ± 4*	31 ± 3	32 ± 5
ylbC	5 ± 4*	0 ± 1*	16 ± 2*	31 ± 0
yqeF	5 ± 2*	4 ± 2*	33 ± 5	28 ± 3
yqhL	16 ± 5	0 ± 1*	34 ± 4	30 ± 1
ytpA	18 ± 2	13 ± 1	30 ± 1	33 ± 1
ytxG	7 ± 5	0 ± 2*	26 ± 2*	29 ± 8
yybT	17 ± 2	0 ± 0*	29 ± 1	36 ± 4

[a] Values are averages and standard deviations of assays on three replicate spore preparations. OD_{600} of purified spore suspension monitored at the indicated time after addition of 1X AGFK or 2xYT while shaking at 37°. * indicates a significant difference from the wild type without P*sspD-gerA* ($p<0.05$).

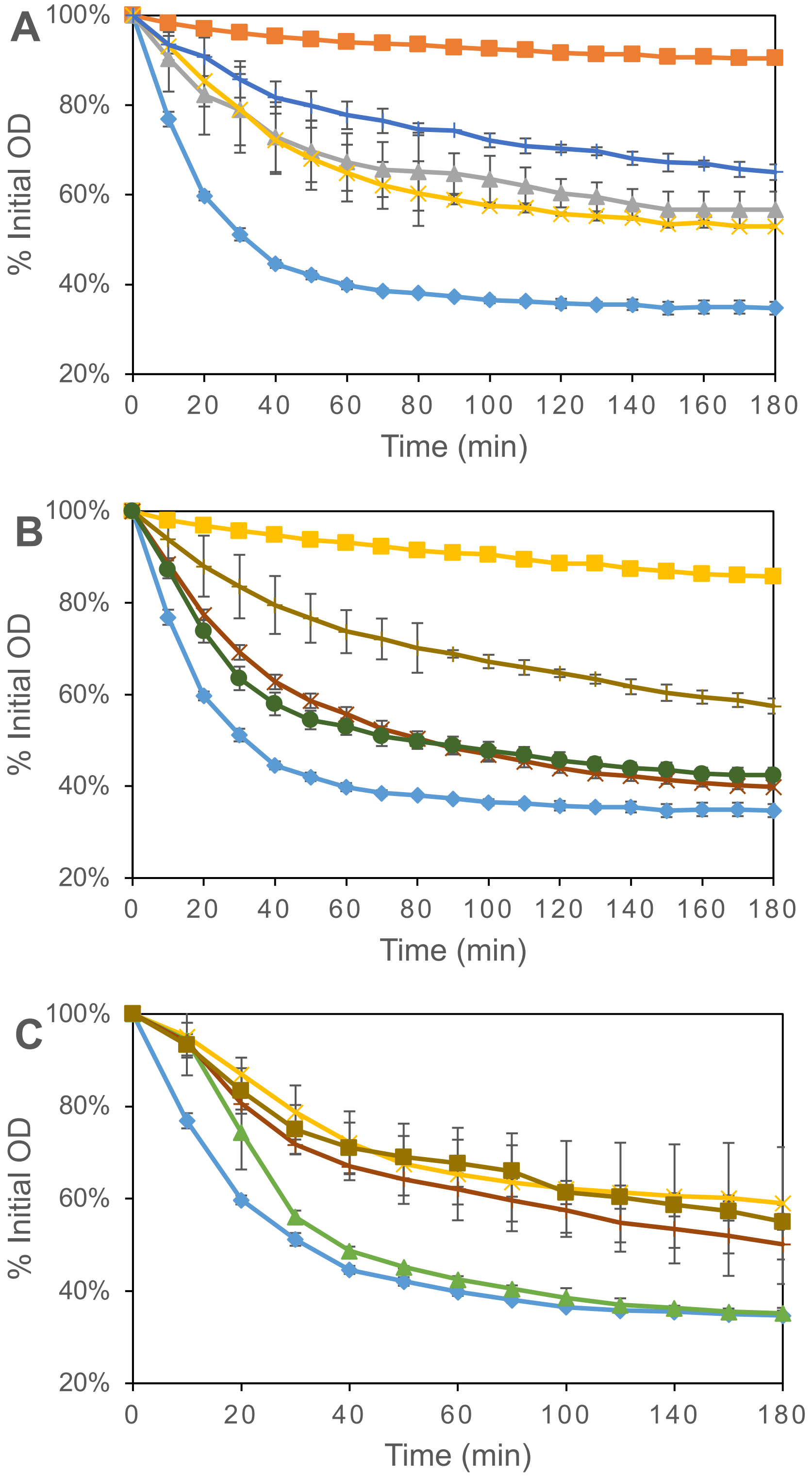

Figure A. Germination rates of *B. subtilis* strains.
Purified spores of *B. subtilis* wild type and mutant strains were heat activated, stimulated to germinate by addition of 10 mM L-Val, and shaken at 37°C, during which the OD_{600} was monitored. Values are averages of three assays and error bars are standard deviations. Each assay was performed on three replicate spore preparations. A) Wild type ♦, *ytxG* **X,** *yqhL* **▲,** *ygaC* **+**, *dnaJ* ■ B) Wild type ♦, *phoR* **X,** *hfq* **+**, *sipT* ■, *yybT* ● C) Wild type ♦, *sfkE* **X,** *pcrB* **▲,** *yqeF* **+**, *ytpA* ■.

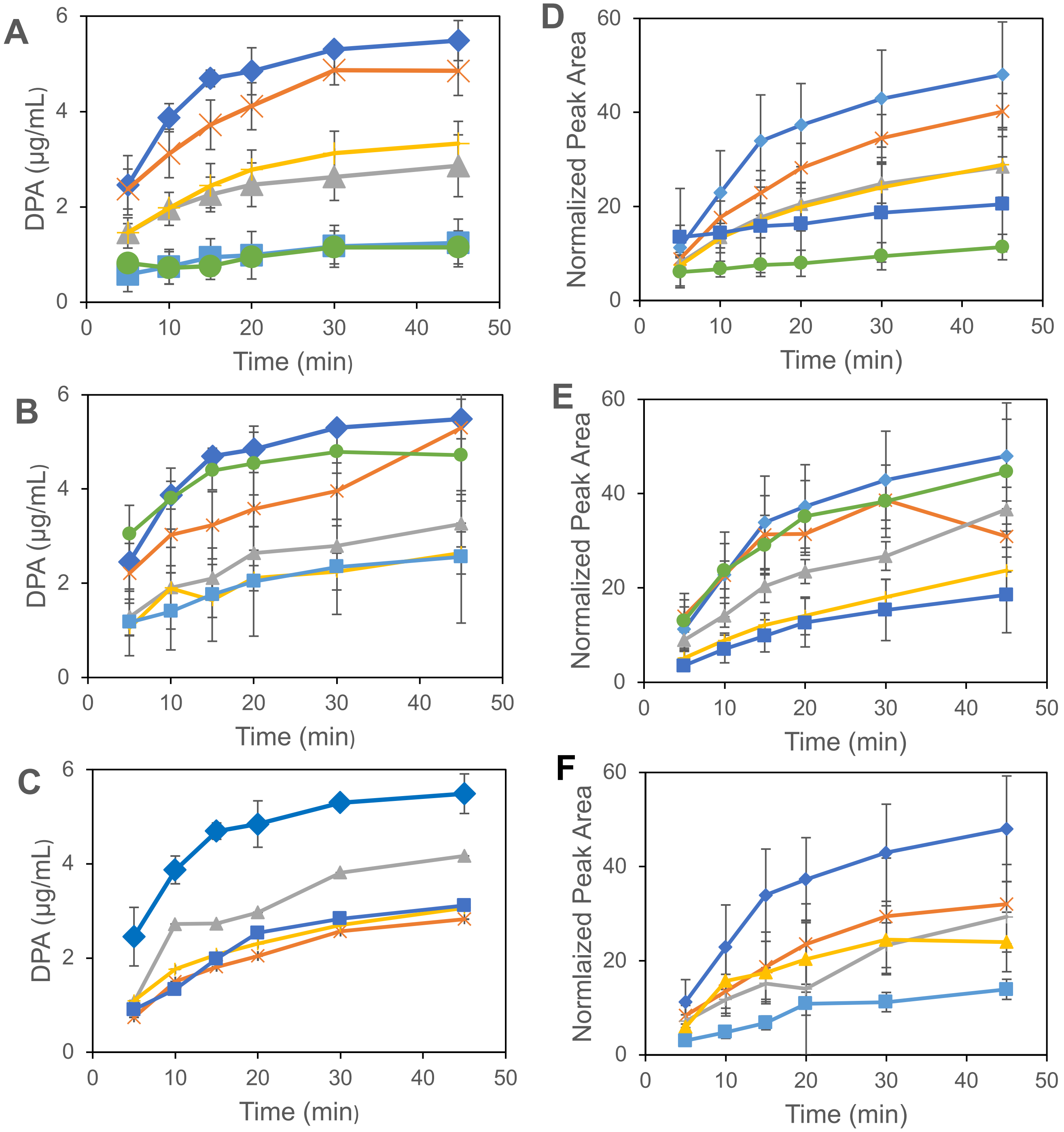

Figure B. Release of DPA and NAM by *B. subtilis* strains.
Purified spores were heat activated, stimulated to germinate by addition of 10 mM L-Val, and shaken at 37°C. Samples were taken at designated intervals, centrifuged, and the supernatant was saved for later analysis. Values are averages of three assays for DPA (panels A-C) and NAM (panels D-F), and error bars are standard deviations. Each assay was performed on three replicate spore preparations. Panels A and D: Wild type ♦, *ytxG* **X,** *yqhL* **▲,** *ygaC* **+**, *dnaJ* ■, *ylbC* ●. Panels B and E: Wild type ♦, *phoR* **X,** *phoP* **▲,** *hfq* **+**, *sipT* ■, *yybT* ●. Panels C and F: Wild type ♦, *sfkE* **X,** *pcrB* **▲,** *yqeF* **+**, *ytpA* ■. For DPA release: *dnaJ, ylbC, yqeF, and ytpA* strains are significantly different from the wild type at all time points; *hfq, phoP, sipT, ygaC,* and *yqhL* strains are significantly different from 10 min onwards; and *pcrB, phoR*, *yybT,* and *ytxG,* strains are not significantly different from the wild type. For NAM release: *sipT* , *ylbC*, and *ytpA* strains are significantly different from the wild type from 20 min onwards. Some other strains exhibited reduced NAM release, but this was not found to be significant due to high variability between replicates.

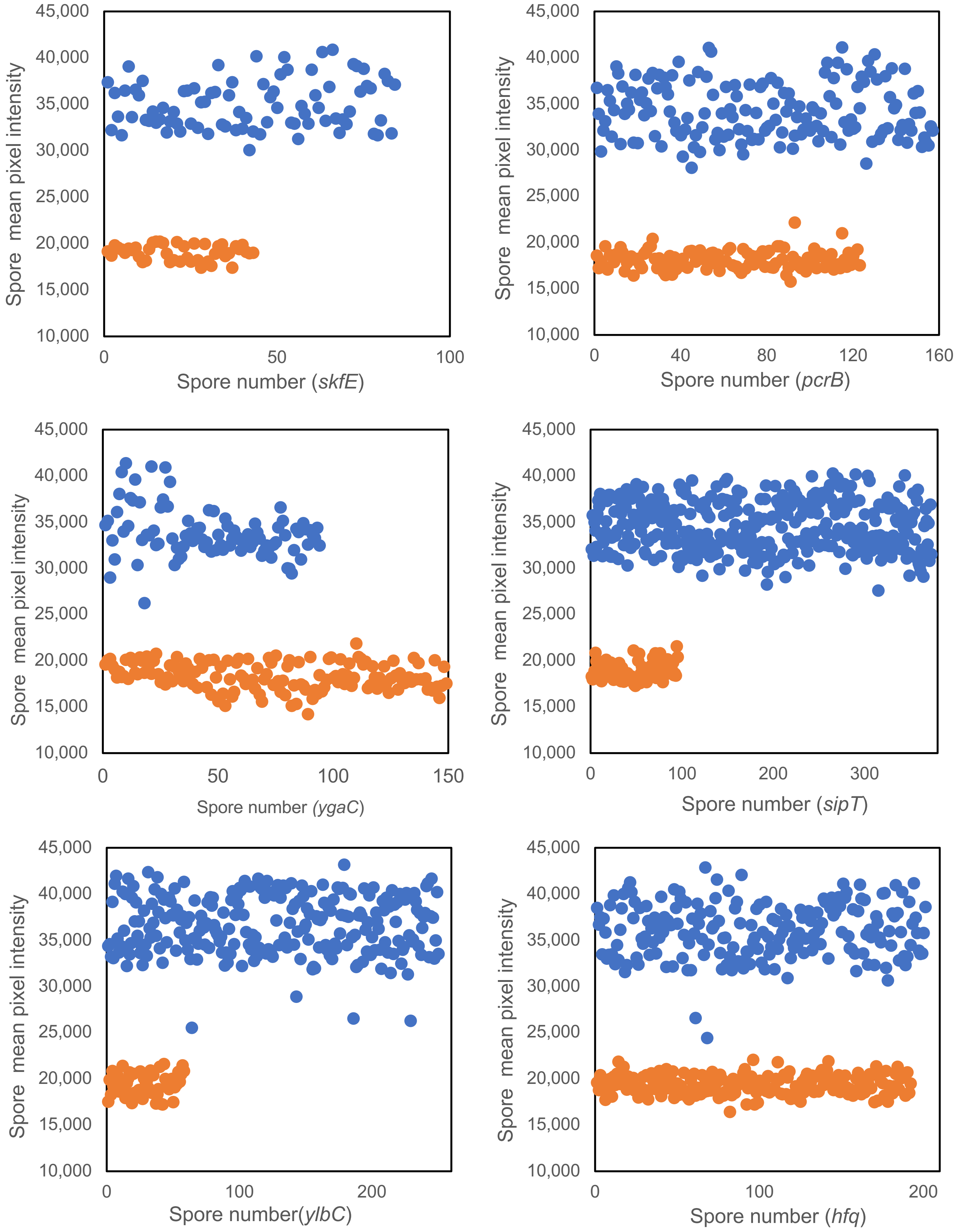

Spore mean pixel intensity
45,000
40,000
35,000
30,000
25,000
20,000
15,000
10,000
0
50
100
Spore number (skfE)
0
40
80
120
160
Spore number (pcrB)
150
Spore number (ygaC)
200
300
Spore number (sipT)
Spore number(ylbC)
Spore number (hfq)

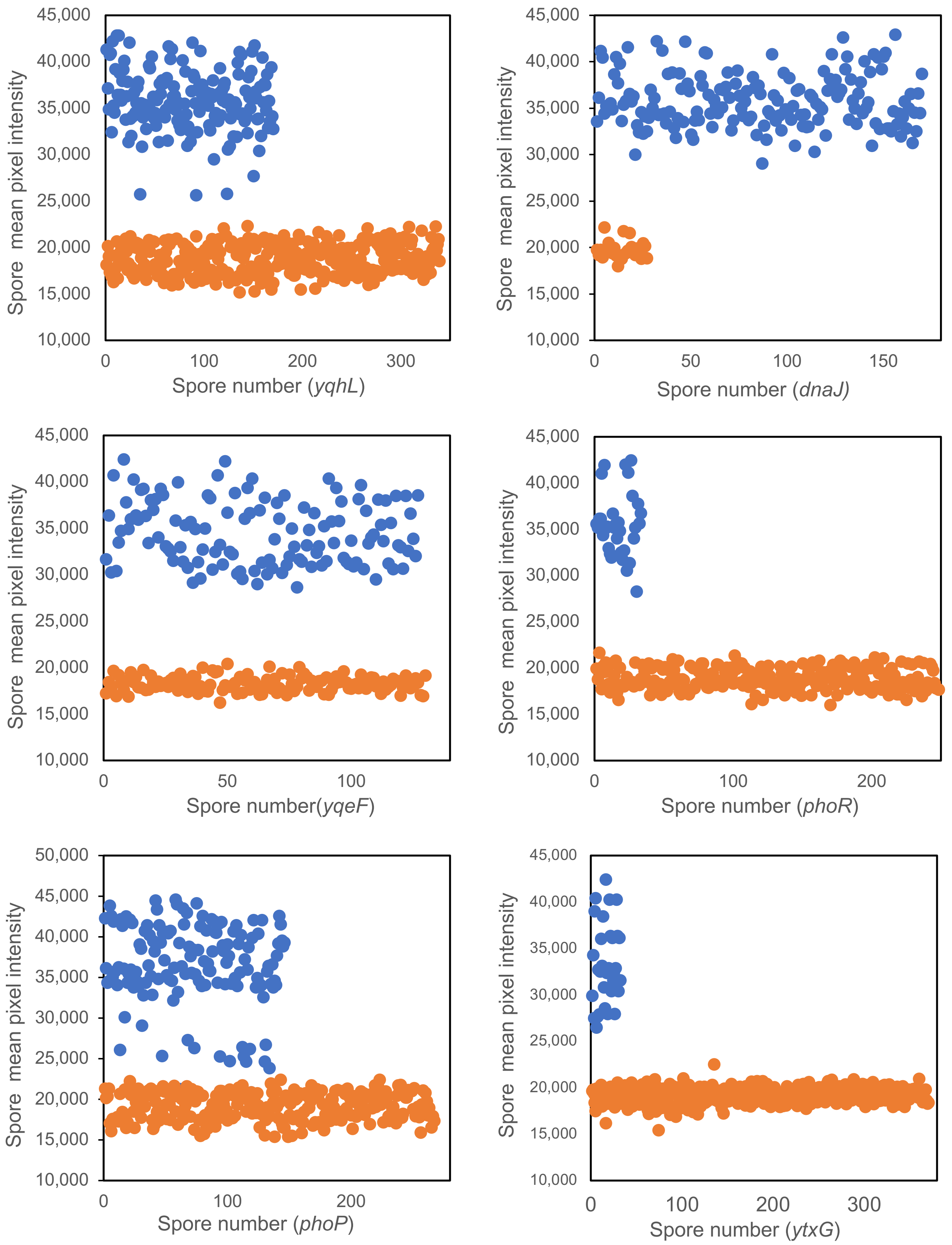
45,000
40,000
35,000
30,000
25,000
20,000
15,000
10,000
Spore mean pixel intensity
0
100
200
300
Spore number (*yqhL*)
45,000
40,000
35,000
30,000
25,000
20,000
15,000
10,000
Spore mean pixel intensity
0
50
100
150
Spore number (*dnaJ*)
45,000
40,000
35,000
30,000
25,000
20,000
15,000
10,000
Spore mean pixel intensity
0
50
100
Spore number(*yqeF*)
45,000
40,000
35,000
30,000
25,000
20,000
15,000
10,000
Spore mean pixel intensity
0
100
200
Spore number (*phoR*)
50,000
45,000
40,000
35,000
30,000
25,000
20,000
15,000
10,000
Spore mean pixel intensity
0
100
200
Spore number (*phoP*)
45,000
40,000
35,000
30,000
25,000
20,000
15,000
10,000
Spore mean pixel intensity
0
100
200
300
Spore number (*ytxG*)

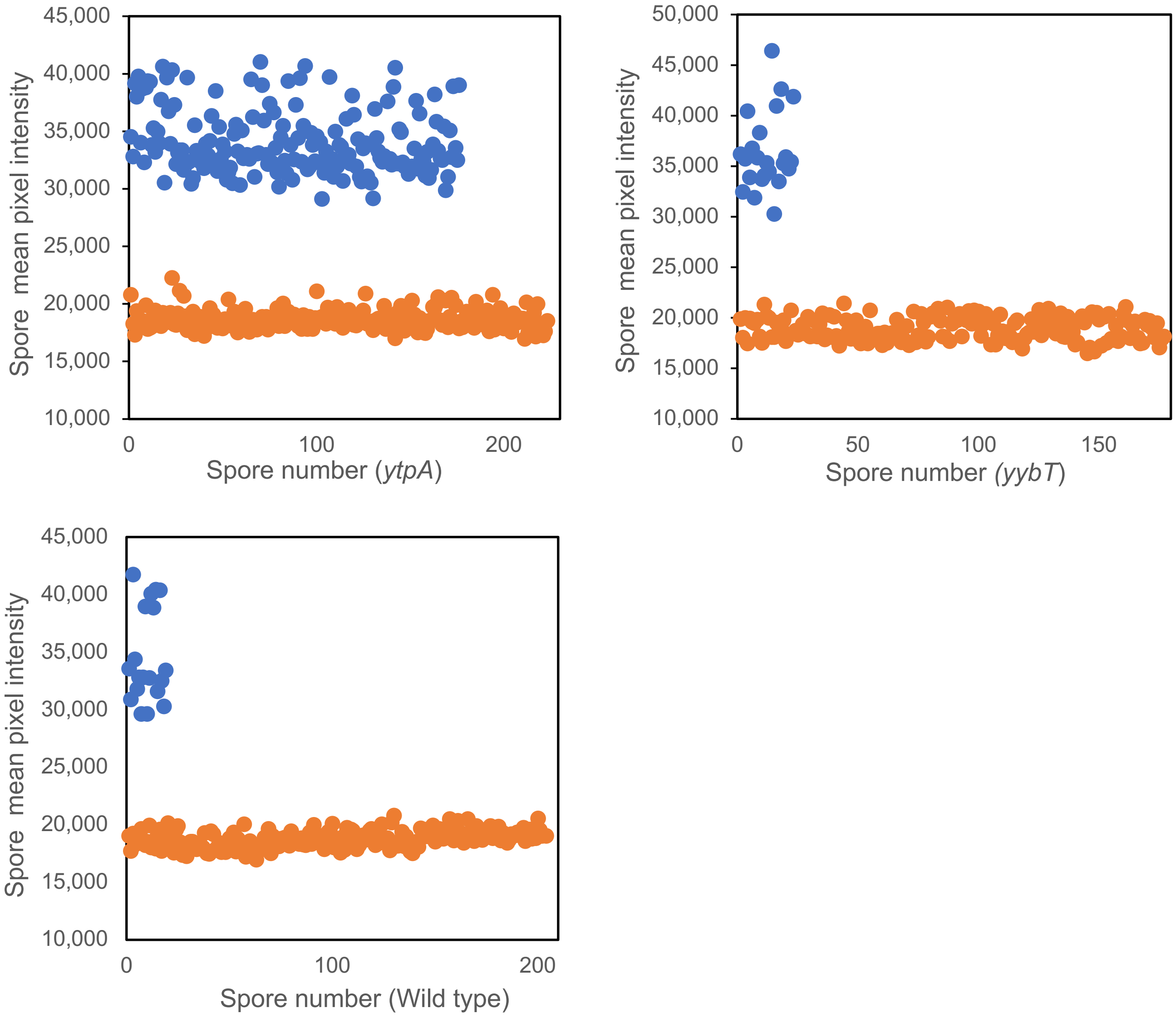

Figure C. Phase contrast microscopy image pixel intensities during spore germination.
Spores were subjected to germination conditions, 10 mM L-Valine at 37°C, for 1 hour. For each strain, pixel intensities were averaged for each spore detected in three images, including a range of 127-509 spores (Each dot represents one spore). Blue dots indicate spores classified as phase bright, which had similar intensities as spores in the initial dormant population, and orange dots indicate spores classified as phase-dark, in order to determine population percentages in Table 3. Phase bright and phase dark spores are plotted independently on the X-axes.

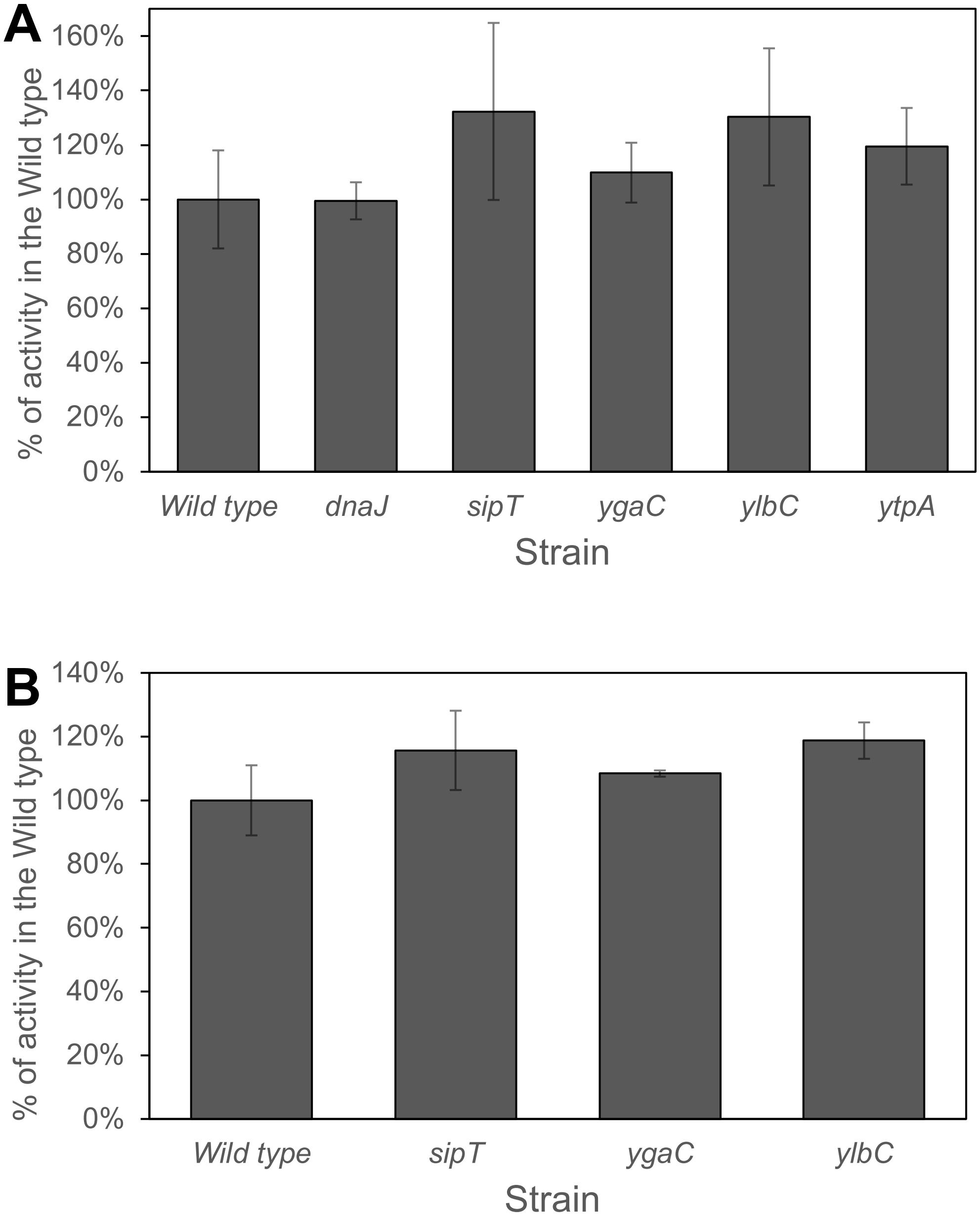

Figure D. Expression of σ^G-dependent genes in *B. subtilis* mutant strains. Purified spores carrying *lacZ* transcriptional fusions were decoated and lysed, and extracts were assayed for β-galactosidase. Values are expressed as a percentage of that detected in the wild type strain containing the same *lacZ* fusion. Values are averages of assays on three (A, *pbpF-lacZ*) or two (B, *sspB-lacZ*) replicate spore preparations and error bars are standard deviations.

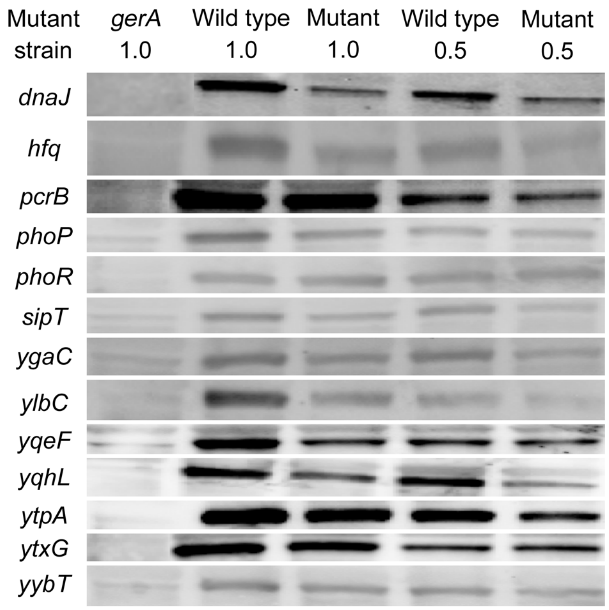

Figure E. GerAC is reduced in the spores of several B. subtilis mutant strains. Equal quantities of spore suspensions were decoated and broken, and proteins were extracted, serially diluted, run on SDS-PAGE, and transferred to PVDF membrane as described previously [5]. The membrane was probed with anti-GerAC antibodies [6]. Strain genotype (All strains were also Δ*gerB*) and sample dilution is indicated above each lane or on the left of each panel.

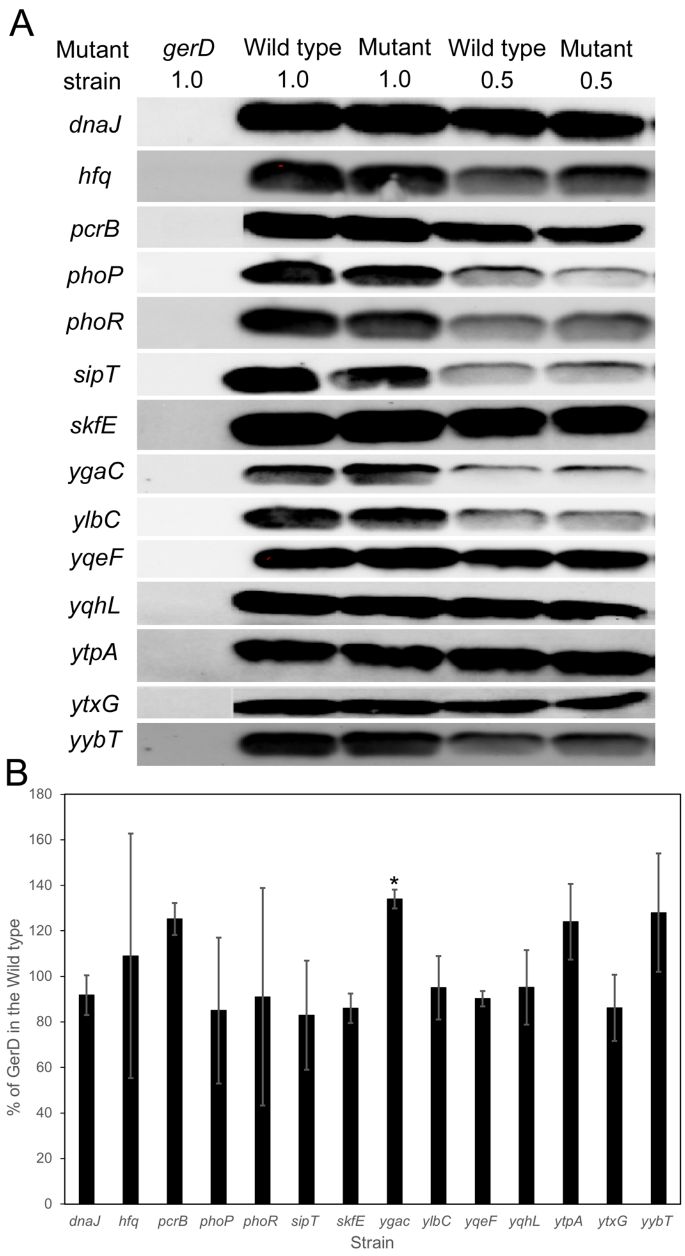

Figure F. GerD is not reduced in the spores of *B. subtilis* germination mutants. Equal quantities of spore suspensions were decoated and broken, and proteins were extracted, serially diluted, run on SDS-PAGE, and transferred to PVDF membrane as described previously [5]. The membrane was probed with anti-GerD antibodies [6] (Panel A). Strain genotype (All strains were also Δ*gerB*.) and sample dilution is indicated above each lane or on the left of each panel. Protein load and transfer to membrane in each lane was normalized as described in Materials and Methods, and the amount of GerD detected in each strain was compared to that found in the wild type (Panel B). Error bars indicate standard deviations. * indicates a significant difference from the wild type ($p \leq 0.05$).

References

1. Paidhungat M, Setlow B, Driks A, Setlow P. Characterization of spores of *Bacillus subtilis* which lack dipicolinic acid. J Bacteriol. 2000;182(19):5505-12. PubMed PMID: 10986255.
2. Feavers IM, Foulkes J, Setlow B, Sun D, Nicholson W, Setlow P, et al. The regulation of transcription of the *gerA* spore germination operon of *Bacillus subtilis*. Molec Microbiol. 1990;4:275-82.
3. Zuberi AR, Moir A, Feavers IM. The nucleotide sequence and gene organization of the *gerA* spore germination operon of *Bacillus subtilis* 168. Gene. 1987;51(1):1-11. Epub 1987/01/01. PubMed PMID: 3110007.
4. Cabrera-Martinez RM, Tovar-Rojo F, Vepachedu VR, Setlow P. Effects of overexpression of nutrient receptors on germination of spores of *Bacillus subtilis*. J Bacteriol. 2003;185(8):2457-64. PubMed PMID: 12670969.
5. Stewart KA, Setlow P. Numbers of individual nutrient germinant receptors and other germination proteins in spores of *Bacillus subtilis*. J Bacteriol. 2013;195(16):3575-82. doi: 10.1128/JB.00377-13. PubMed PMID: 23749970; PubMed Central PMCID: PMC3754565.
6. Ramirez-Peralta A, Zhang P, Li YQ, Setlow P. Effects of sporulation conditions on the germination and germination protein levels of *Bacillus subtilis* spores. Appl Environ Microbiol. 2012;78(8):2689-97. Epub 2012/02/14. doi: 10.1128/AEM.07908-11. PubMed PMID: 22327596; PubMed Central PMCID: PMCPMC3318806.
